# Inflammatory bowel disease patient perceptions of diagnostic and monitoring tests and procedures

**DOI:** 10.1186/s12876-019-0946-8

**Published:** 2019-02-13

**Authors:** Isabelle Noiseux, Sophie Veilleux, Alain Bitton, Rita Kohen, Luc Vachon, Brian White Guay, John D. Rioux

**Affiliations:** 1 Department of Management, Université Laval, Quebec, G1V 0A6 Canada; 2 Division of Gastroenterology, McGill University Health Centre, Montreal, H3A 0G4 Canada; 3 iGenoMed Consortium, Montreal, H1T 1C8 Canada; 4 Department of Family Medicine and Emergency Medicine, Université de Montréal, Montreal, H3T 1J4 Canada; 5 Department of Medicine, Université de Montréal & Montreal Heart Institute, Montreal, H1T 1C8 Canada

**Keywords:** Monitoring, Diagnostic, Inflammatory bowel disease, Tests, Procedures

## Abstract

**Background:**

Inflammatory Bowel Disease (IBD) with its high incidence and prevalence rates in Canada generates a heavy burden of tests and procedures. The purpose of this study is to gain a better understanding of the transfer of information from physician to patient, as well as the patient understanding and perceptions about the tests and procedures that are ordered to them in the context of IBD diagnosis and monitoring.

**Methods:**

An online questionnaire was completed by 210 IBD patients in Canada. Information on the five most-often used tests or procedures in IBD diagnosis/monitoring was collected. These include: general blood test, colonoscopy, colon biopsy, medical imaging and stool testing.

**Results:**

The general blood test is both the most ordered and most refused tool. It is also the one with which patients are the least comfortable, the one that generates the least concern and the one about which physicians provide the least information. The stool test is the test/procedure with which patients are the most comfortable. Procedures raise more concerns among patients and physicians provide more information about why they are needed, their impact and the risks they present. Very little information is provided to patients about the risks of having false positives or negative tests.

**Conclusions:**

This study provides an initial understanding of patient perceptions, the transfer of information from a physician to a patient and a patient’s understanding of the tests and procedures that will be required to treat IBD throughout what is a lifelong disease. The present study takes a first step in better understanding the acceptance of the test or procedure by IBD patients, which is essential for them to adhere to the monitoring process.

**Electronic supplementary material:**

The online version of this article (10.1186/s12876-019-0946-8) contains supplementary material, which is available to authorized users.

## Background

Inflammatory bowel disease (IBD) includes ulcerative colitis (UC) and Crohn’s disease (CD). These are chronic inflammatory illnesses of the gastrointestinal tract of unknown etiology. There is no cure, and the purpose of treatment is to control the symptoms and maintain remission [1, 2]. Canada ranks among the countries with the highest prevalence and incidence rates of IBD in the world [3]. Furthermore, Canada has one of the highest IBD incidence rates among the under 16 age group and this rate increasing, especially among children under 5 years of age [4]. One out of every 150 Canadians is afflicted with IBD [5]. This burden generates a significant economic weight. In 2012, IBD-related costs were estimated at $2.8 billion, of which $1.2 were direct costs (hospitalization, medication and medical visits) [1].

Diseases for which diagnosis, monitoring, and surveillance are appropriate are those that significantly impact a person’s quality of life, are fatal, and are sufficiently widespread to justify investments in conditions for which early detection is beneficial and for which treatment exists [6, 7]. IBD meets all of these criteria. The management of IBD patients requires assessment both at the time of diagnosis and throughout the illness to determine the activity and severity of the inflammatory lesions, disease location, progression and complications [8]. In chronic illness it has been reported that, due to time constraints on the part of the physician, patients do not receive the care they require and, as a result, their illness remains unmonitored [9, 10]. On the other hand, it has also been reported that patients undergo too many tests and procedures. Some authors [11, 12] have put forward the suggestion that this overuse of tests and procedures can be explained by the fact that physicians receive a bonus for each test requested or that they do more than less out of fear of lawsuits, which can lead to false positives or overdiagnoses.

IBD diagnosis and monitoring are mainly based on an in-depth physical examination coupled with the patient’s medical history and various tests and procedures that include blood tests, stool tests, endoscopy with or without biopsy and medical imaging [13]. The clinical signs of UC include urgency, tenesmus, bloody diarrhea or abdominal pain. Signs of CD are more variable and dependent on the extent and the location of the gastrointestinal disease and on whether or not there are complications such as intestinal strictures, intestinal or perianal fistulas or abscesess [14]. Periodic measures including office visits, laboratory tests and procedures are part of the monitoring process that helps manage chronic illnesses [15]. Assessment of IBD activity is mainly carried out through symptom reporting, laboratory testing and endoscopy. As example, a fecal calprotectin test can be used to monitor disease activity. In addition, for CD radiologic imaging plays an important role in assessment of disease activity. [16]. The ideal test must be safe, simple, inexpensive, acceptable to the public and must also be reproducible, sensitive and specific [6, 16]. IBD patients are subject to a large quantity of medical care, tests and procedures [17–20].

The various tests and procedures to diagnose and monitor IBD all have specific goals. Blood tests are used to screen for IBD and assess a patient’s state of health [18]. Repeated measures of certain biomarkers such as calprotectin or lactoferrin in the stool are part of the clinical IBD management procedures [17, 21]. These biomarkers allow for quick and non-invasive monitoring of inflammation [22, 23]. Endoscopy plays an integral role in the diagnosis and management of IBD patients. An endoscopic examination allows physicians to distinguish CD from UC and also provides information on disease extent and severity [24]. Patients with IBD, are at higher risk of developing colorectal cancer (CRC), which is monitored through endoscopy with biopsies [25, 26]. Medical imaging including ultrasound, CT Scan and Magnetic Resonance Imaging are performed in IBD patients as a diagnostic tool and to determine the extent of the damage to the intestines, monitor the illness’ activity and assess for complications [19, 27]. Certain tests, such as a colonoscopy or blood tests, are not appreciated by patients and generate anxiety [28–32]. It is therefore important that patients receive and understand the information about the risks and benefits of the various tests and procedures [33, 34].

Despite improvements in the available treatment options, IBD continues to have a negative impact on the quality of life of patients [35]. Many studies have focused on the impact of IBD on quality of life [36, 37], the need for information [38], strategies to adapt [39] and shared decision making [40]. Despite the funding and resources invested in the diagnosis and monitoring of IBD, few studies have focused on these activities. To the best of our knowledge, no studies have been conducted on the perception of patients toward diagnostic tests and monitoring specifically for IBD patients. Even less information is available on patient understanding of and compliance with the tests and procedures that are requested by their physicians. Yet, the literature shows that understanding how a chronic condition influences patients and their ability to adhere to health care recommendations is essential, especially as part of a patient-centered approach [41]. Questions to be posed include: What are the percentages of orders for tests and/or procedures? What percentages of patients refuse these tests/procedures and for what reasons? What information is given to the patient about these tests? What is the patient’s understanding of these tests? Do these tests generate any concerns for the patient?

The purpose of this study is to gain a better understanding of the transfer of information from physician to patient, as well as patient understanding and perceptions of the tests and procedures that are ordered by their doctor in the specific context of IBD diagnosis and monitoring.

In a patient-centered approach, it is essential to gain a better understanding of patient perceptions of the diagnostic and monitoring tests and procedures used [42] in the context of the chronic illnesses that are IBD. This study therefore aims to take a first step in this regard. With an increased awareness of the problems associated with the tests and procedures, physicians can prevent these negative perceptions and adapt their exchange of information with their patient, which will in turn soften the impact of the tests and procedures on the quality of life of their patients.

## Methods

The current study was part of a larger research program aimed at translating genetic discoveries into a personalized approach for the treatment of inflammatory bowel diseases [48] for which an online questionnaire was specifically developed. One of the sections of this questionnaire was designed to better understand the concerns raised by the tests/procedures among IBD patients, the transfer of information from the physician to patients and the patients’ understanding of these tests/procedures, as well as the rate of prescriptions, the reasons patients refuse to undergo such tests/procedures and the sociodemographic profile of the participants (Additional file 1: Online Questionnaire).

The survey was posted on the website of Crohn’s and Colitis Canada (CCC), an association that has 933 patient members, and which made it possible to reach patients in a manner that respected their privacy. Patients could access the survey through the CCC website over a 5-month period. Five reminders were posted on the CCC website, newsletter and social media via existing CCC platforms in an attempt to reach the largest possible final sample size. In total, 210 adult participants were reached across 10 different Canadian provinces and/or territories, for a response rate of 22.5%, which is within the range previously reported in similar studies [43–45]. The vast majority of the 210 respondents answered all of the questions.

The questionnaire was built in five sections to collect information on the five most used tests and procedures for the diagnosis and monitoring of IBD: general blood test, stool test, colonoscopy, colon biopsy and medical imaging. For each section, patients were asked to answer 20 questions to assess their acceptance or refusal of a given test/procedure, the reasons for the refusal, their concerns, the level of comfort with undergoing the test (confort is defined by: the patient feel relaxed and wellbeing toward those tests, have no negative perception and perceive them relatively free from pain), their understanding, and the information provided by their physician in connection with every test or procedure under study. Respondents indicated their agreement or disagreement with a given statement on a Likert scale [46]. This type of scale was chosen as it makes it possible to measure complex attitudes or individual perceptions. An even-numbered scale [6] was used, as it eliminates the respondents’ tendency to choose the middle answer, known as central tendency [47]. The online questionnaire was first “pre-tested” by 14 IBD treatment experts and then by 17 patients in a gastroenterology clinic in order to check their understanding of each question. Minor changes in the wording were subsequently made to complete the final questionnaire. All participants signed the information and consent form before completing the questionnaire. The questionnaires were completed anonymously; the respondents cannot be tracked. The information and consent form was approved by the ethics committee (approval number 2013–041 / 27-09-2013).

This study presents the analysis of the responses obtained regarding the patients’ laboratory tests and procedures. SPSS statistical software was used for calculations. Pearson’s chi-square test (χ2) was use to evaluate the correlation between educational and understanding level. The Likert scale was grouped into low [1, 2], medium [3, 4] and high [5, 6] response categories for effects to emerge more clearly.

## Results

### Prescriptions and refusal to undergo tests or procedures

The sociodemographic profile of the entire population under study (*n* = 210) is presented in Table 1. More women (171) than men [39] participated to the web survey. Among the patients who anwered the questionnaire, there were more patients having Crohn’s disease (145) than ulcerative colitis (65), more patients in the age range 18–34 (82) than in the age range of 35–44 (68) and the less represented age range was 45 and over (56). The educational level of patients who answered the questionnaire were grouped in high school [34], professional or college (85) or university diploma (87). The distribrution of the province or territory where were living the patients were living is also presented in Table 1. Ontario was the province from which the highest number of patients participated in the web questionnaire while Nunavut was the least represented territory.

**Table 1:** Sociodemographic profil of the entire population under study (*n* = 210)

Sociodemographic characterisitcs		*n*
Gender	Men	39
	Women	171
Type of Inflammatory bowel disease	Ulcerative Colitis	65
	Crohn’s disease	145
Age	18–34	82
	35–44	68
	45 and over	56
Educational level	High school	34
	Professional or college	85
	University diploma	87
Province of residence among Canada	Alberta	15
	British-Colombia	14
	Manitoba	3
	New-Brunswick	7
	Nova-Scotia	13
	Nunavut	1
	Ontario	84
	Quebec	61
	Saskatchewan	5
	Newfoundland and Labrador	7

With regard to diagnosis or monitoring, the incidence of tests and procedures requested for the entire population under study (*n* = 210) and the number of tests and procedures that were refused by these patients are presented in Table 2. The most-often ordered test/procedure was a blood test (96.7% of patients), followed by a colonoscopy (93.3%), a colon biopsy (81.4%), a stool test (67.1%) and the least-often requested procedure was medical imaging (58.1%). Some patients decided to refuse to undergo these tests/procedures. The rate of refusal is similar for the majority of the tests/procedures (2 to 5 refusals), but is significantly higher for the blood test, which was rejected by 74 patients. Therefore, although a blood test is the most-often ordered test, it is also the one that is the most often refused.

**Table 2:** Number of Tests/Procedures Requested and Refused by Inflammatory bowel disease Patients (*n* = 210)

	Test/procedure requested		Refused the test/procedure	
	*n*	%	*n*	%
General blood test	203	96.7	74	36.5
Colonoscopy	196	93.3	2	1.0
Colon biopsy	171	81.4	2	1.2
Medical imaging	122	58.1	5	4.1
Stool test	141	67.1	4	2.8

The reasons why patients refused to undergo the tests and procedures are presented in Table 3. Time, pain, costs, potential risks, side effects, fear of results, test too revealing, and confidentiality were reported by patients as reasons for refusal. None of the suggested reasons explained why 4 patients had refused the stool test and why 5 patients had refused the medical imaging. On the other hand, a colonoscopy was refused by 2 patients, at least one of whom had refused the procedure for several of the reasons suggested: time, pain, potential risks, side effects, fear of results, test too revealing and confidentiality. The colon biopsy was also refused by 2 patients, at least one of whom refused the test because of the potential risks. The blood test, which was refused by 74 patients with the reasons reported in the questionnaire only partly explained the reasons for the refusal. Sixty-five patients replied that the reason they had refused the test was not listed in the questionnaire. Other patients refused the blood test for the following reasons suggested in the questionnaire: time, pain, costs, potential risks, side effects, fear of results, test too revealing and confidentiality.

**Table 3:** Reasons for a Patient to Refuse to Undergo the Requested Test/Procedure

Reason to refuse to undergo the test/procedure		General blood test (*n* = 74)	Colonoscopy (*n* = 2)	Colon biopsy (*n* = 2)	Medical imaging (*n* = 5)	Stool test (*n* = 4)
Time	Not at all for this reason	67	1	2	5	4
	Partially for this reason	3	0	0	0	0
	Refused for this reason	4	1	0	0	0
Pain	Not at all for this reason	68	1	2	5	4
	Partially for this reason	1	0	0	0	0
	Refused for this reason	2	1	0	0	0
Costs	Not at all for this reason	66	2	2	5	3
	Partially for this reason	5	0	0	0	0
	Refused for this reason	1	0	0	0	0
Potential risks	Not at all for this reason	69	1	1	5	4
	Partially for this reason	2	0	0	0	0
	Refused for this reason	1	1	1	0	0
Side effects	Not at all for this reason	69	1	1	5	4
	Partially for this reason	2	0	0	0	0
	Refused for this reason	1	1	1	0	0
Fear of results	Not at all for this reason	65	1	2	5	4
	Partially for this reason	3	0	0	0	0
	Refused for this reason	3	1	0	0	0
Test too revealing (fear of finding other problems)	Not at all for this reason	68	1	1	5	3
	Partially for this reason	1	1	0	0	1
	Refused for this reason	2	0	0	0	0
Confidentiality	Not at all for this reason	70	1	2	5	4
	Partially for this reason	0	1	0	0	0
	Refused for this reason	1	0	0	0	0

### Patient perceptions of the impact of the tests and procedures

The level of comfort with the test or procedure was determined by the patients. Level of comfort referring to feeling relaxed and wellbeing toward those tests, have no negative perception and perceive them relatively free from pain. Although initially rated from 1 to 6 on the Likert scale, the level of comfort was grouped into three levels to more easily highlight the trends: low (rating of 1–2), medium (rating of 3–4) and high (rating of 5–6) (see Table 4). Comfort level was lowest for the blood test, where 78.9% of patients reported a low level of comfort, whereas 9.8% of patients felt comfortable with this test. The second lowest level of comfort was for the colonoscopy, for which 40.8% of patients expressed a low level of comfort and 24.8% appeared to be comfortable with this procedure. Among the tests/procedures included in our study, patients were most comfortable with the stool test, where only 12.2% expressed a low level of comfort and 61.4% expressed a high level of comfort with this test. Patients also appeared to be comfortable with the medical imaging and the colon biopsy, with percentages of high levels of comfort that were similar to those reported for the stool test: 60.8 and 54.1% respective.

**Table 4:** Impact of Test/Procedure on Patient Level of Comfort and Concerns

		General blood test	Colonoscopy	Colon biopsy	Medical imaging	Stool test
Level of comfort with this test	Low	78.9	40.8	18.3	12.5	12.2
	Medium	11.3	34.7	27.6	26.7	26.4
	High	9.8	24.5	54.1	60.8	61.4
Level of concern when results are presented	Low	35.4	10.2	19.4	18.2	28.5
	Medium	45.1	30.1	41.2	43.8	42.3
	High	19.5	59.7	39.4	38.0	29.2
Results increase the concerns about the disease	Yes	17.8	64.3	48	52.1	37.7
	No Results stable	52.4 29.8	35.7	52	47.9	62.3

Patients also assessed their levels of concern during their specialists’ presentation of the results of the tests/procedures (see Table 4). Level of concern was lowest for the results of the blood test, where 19.5% of patients reported a high level of concern. For the colonoscopy, 59.7% of the patients reported a high level of concern upon receiving the results. The patients were also concerned about the results of the colon biopsy and the medical imaging, but to a lesser degree, with 39.4 and 38% respectively reporting a high level of concern.

Patients then assessed the impact of these test/procedure results on their concerns about their illness (Table 4). The colonoscopy generated the greatest increase in concern about the illness (64.3%). Next were the medical imaging results (52.1%), followed by the colon biopsy (48%) and the stool analysis (37%). The blood test had the least impact on patient concerns about their illness (only 17.8% of respondents reported an increase in their concerns).

### Patient understanding of the tests and procedures

The patients were asked whether their specialist had explained the reason why the test/procedure had been requested (Table 5). A total of 95.5% of patients had received explanations as to why the colonoscopy had been requested; 87.9% for the medical imaging; 83.2% for the colon biopsy; 78.7% for the blood test; and 73.2% for the stool test. The patients were also asked about their understanding of the reason why the test/procedure was requested. The highest level of understanding of the reason why the test/procedure was needed was for the colonoscopy (86.9%), followed by the stool test (76.1%), the medical imaging scan (75%), the colon biopsy (71.1%) and, lastly, the blood test (63.5%). Pearson’s chi-square test (χ2) was performed to evaluate the correlation between educational level and the level of understanding of the reason why the test/procedure was requested. For every tests and procedures, no significant correlation between educational level and understanding was found. Patients then reported their level of understanding of the potential treatments in connection with the test/procedure they had undergone. The highest level of understanding was reported for the colonoscopy with only 59.4%, followed by the stool test (53.9%), the colon biopsy (48.3%), the blood test (47.8%) and the medical imaging scan (45.1%). For this question, the level of understanding was not very high, as all were reported below 60%.

**Table 5:** Patient Understanding of the Tests and/or Procedures

		General blood test	Colonoscopy	Colon biopsy	Medical imaging	Stool test
Did the physician explain why this test was requested?	Yes	78.7	95.5	83.2	87.9	73.2
	No	21.3	4.5	16.8	12.1	26.8
What is your level of understanding of the reason why the test/procedure was requested?	Low	10	0.5	7.0	8.1	6.3
	Medium	26.5	12.6	21.9	16.9	17.6
	High	63.5	86.9	71.1	75.0	76.1
What is your level of understanding of the potential treatments?	Low	12.5	8.1	13.4	15.4	12.1
	Medium	39.8	32.5	38.3	39.5	34.0
	High	47.8	59.4	48.3	45.1	53.9

Table 5 presents the percentage of patients whose physician had explained why the test/procedure was requested. For all tests and procedures, other than the stool test, there is a decrease in the percentage of patients who had a high understanding of why the test was requested from those who had received the information from their physician. Therefore, even though a certain percentage of patients received explanations as to why the tests/procedures were requested there was therefore a loss of understanding. This loss of understanding was even more pronounced when patients were asked about their level of understanding of the potential treatments. For example, 78.7% of patients received explanations as to why the blood test was requested. Among these patients, only 63.5% had a high understanding as to why it was requested, and only 47.8% of the patients understood the potential treatments in connection with the tests/procedure. This was the case for all tests/procedures other than the stool test. Although 73.2% of patients received explanations of the reason this test was requested, 76.1% of patients had a high understanding of why it was requested, and 53.9% of patients understood the potential treatments. Therefore, for the stool test, the information appears more intuitive. However, for the other tests/procedures, although the information was given to the patients, it does not appear to have been fully understood.

### Transfer of information about the tests and procedures

Patients were asked if the specialist had informed them of the impact the results of the tests/procedures would have on treatment options; the possibility of false positives or negatives; any potential risks; and the level of invasiveness (for procedures only) (see Table 6). Patients were therefore asked whether the physician had informed them of the impact of the results of the test/procedure on treatment options. Most patients reported having received this information about the colonoscopy (83.2%), followed by the stool test (73.2%), the colon biopsy (72.7%) and the medical imaging (72.6%), the latter three being very similar. Patients reported having received the least information about the impact of the results of blood tests on treatment (59.9%). Information about the possibility of false positives or negatives was the least shared with patients. A total of 40% of patients were informed about possible false positives or negatives for the stool test, which is the highest percentage, closely followed by the medical imaging scan (36.6%), the colon biopsy (34.7%) and the colonoscopy (32.3%). Only 22.3% of patients were informed about possible false positive or negative results of blood tests.

**Table 6:** Information Provided by the Physician Concerning the Test/Procedure

Did the Inflammatory bowel disease specialist inform you:		General blood test	Colonoscopy	Colon biopsy	Medical imaging	Stool test
Of the impact that each result may have on the treatment?	Yes	59.9	83.2	72.7	72.6	73.2
	No	40.1	16.8	27.3	27.4	26.8
That there may be a false positive, false negative?	Yes	22.3	32.3	34.7	36.6	40
	No	77.7	67.7	65.3	63.4	60
Of the potential risks?	Yes	23.4	73.2	68.4	60.5	47.5
	No	76.6	26.8	31.6	39.5	52.5
On the level of invasiveness?	Yes	NA	75.6	71.1	63.4	NA
	No		24.4	28.9	36.6	

Patients were also asked whether they had received information about the potential risks of the test/procedure. Information about potential risks was most often given to patients about the colonoscopy (73.2%), followed by the colon biopsy (68.4%), the medical imaging scan (60.5%) and the stool test (47.5%). Patients were the least informed about the potential risks of blood tests. Lastly, patients were asked if they had been informed about the level of invasiveness of the procedure. A total of 75.6% of patients had been informed of the level of invasiveness of the colonoscopy, and the medical imaging scan was the procedure about which the smallest percentage of patients received this information (63.4%).

## Discussion

The present study aims to gain a better understanding of the concerns generated by tests and procedures; the transfer of information from the physician to the patient; the patients’ understanding of these tests/procedures; as well as the rate of prescription and the reasons that lead patients to refuse to undergo these tests/procedures.

With respect to the percentages of patients who had received the information from their physician, respectively for each test/procedure, there is a decrease in the percentage of patients with a high level of understanding of the reasons why the tests/procedures were requested. Thus, from the number of patients who had received explanations of the request for a test, fewer patients reported understanding as to why the test was requested, other than for the stool test. The stool test may be messy and most inconvenient because the patient often has to return to the clinic with the specimen. But if the patient understands the value in the stool test to rule out infection or to assess for inflammation, then this may explain why the patient has a natural high understanding of this test. No correlation was found between educational level of patients and their understading of the reason why the test was requested. These results are consistent with the literature, since knowledge acquisition is a complex cognitive process that involves learning, communication and reasoning [2]. It has been shown that information comprehension is strictly rational and based on the quality of the transfer of information [48]. Asking patients whether they understand why the test/procedure was requested makes it possible to check whether they have a rational understanding of the information.

This loss of understanding is all the more pronounced when patients were asked to assess their level of understanding of the potential treatments, respectively for each test/procedure. The link between undergoing a test/procedure and the impact on the potential treatment affects the patient on a personal level and implies that he or she must decide whether or not to undergo the test/procedure, the results of which will have a repercussion on the potential treatments. The integration of concepts involving technical medical information, health risks and probabilities, which are difficult concepts to process for patients not accustomed to this type of discourse, can be overwhelming, especially when fear comes into play. When patients are faced with complex information that involves making a decision, their ability to apply their values and principles may be hindered by their emotions or cognitive interference [12]. In such cases, the processing of information and the related decisions are based on more than a rational understanding of the information about the risks, benefits and uncertainty, as the decisions must align with the patient’s fears, preferences and values. As has been previously observed, the quality of the transfer of information appears to have a direct effect on patient involvement, without being strongly mediated by the rational understanding of the information [48]. Thus, understanding why a test/procedure is requested is rational, but understanding the link with the potential treatment calls into play a degree of emotion and cognitive interference that alters the way the patient processes the information, which may translate into a loss of understanding.

In this study, it has been shown that the stool test was the test/procedure for which the risk of false positives or negatives was most often explained, with only 40% of patients receiving this information. Yet, this risk is real for each of the tests/procedures. A good test may come up normal with patients with the illness (false negatives), and may also come up positive with patients who are not ill (false positives). A potential danger of monitoring is getting false positives and the related consequences such as morbidity, unnecessary additional diagnostic tests, invasive procedures and exposure to radiation [6]. On the other hand, a lack of sensitivity and specificity can result in complications being missed, resulting in patient decline that could otherwise have been avoided. These are the reasons why physicians should take greater care to properly explain the risks of obtaining false positives or negatives to patients on the basis of the tests and procedures the patient will have to undergo.

Among the five tests and procedures used to diagnose and monitor IBD, the general blood test is the most ordered, but also the most refused by patients. This is the test/procedure with which patients are the least comfortable. The blood test is also the test that generates the least concern about the results, and the results of which generate the least concern about the illness. The blood and stool tests are the tests/procedures for which explanations about their necessity are provided the least often. The use of blood tests is very common, especially for patients with chronic illnesses, but this test is linked to a fear of needles, which can have serious consequences leading to non-adherence and avoidance of health care [30]. The severe form of this fear is a phobia that is characterized by an intense and irrational fear of blood, needles, medical care and injuries [28]. A fear of needles affects between 14 and 38% of the adult population, whereas the prevalence of the phobia lies in the 3 to 4.5% range [31]. The findings of the study presented here are consistent with these data. A total of 36% of patients (74 patients) refused to undergo a blood test. Among them, only 9 refused for reasons such as time, pain, costs, potential risks, side effects, fear of results, test too revealing or confidentiality. Thus, the remaining patients refused the blood test for reasons other than those suggested in the questionnaire. It is therefore possible that the fear of needles and blood be the main reason for refusing to undergo the blood test [30]. It is also possible that general blood test have been already performed before the patient was referred to the gastroenterologist specialist, explaining that the patient refuse to do it again. The frequency of the blood testing which was requested may explain patients’ low level of comfort and compliance. Six patients mentioned cost as a reason for refusal. Since these tests are mostly covered by the public health system in Canada, future studies could further explore the costs to patients for blood testing. Additional studies would be required to better understand the reasons for refusing the blood test as part of the IBD monitoring process.

For its part, the stool test ranks among the least ordered test/procedure and few patients refuse this test. This is the test with which patients feel the most comfortable. As with the blood test, concern about the results was low and the results did not generate too many concerns about the illness. Various laboratory tests are used in screening patients with suspected IBD. These tests make it possible to identify IBD patients who are relapsing or at risk of relapse [22]. In the past, laboratory markers were underestimated due to their low specificity. Given that endoscopic assessment is invasive and requires significant resources, identifying biomarkers of an illness’ activity becomes an attractive alternative [23]. Fecal biomarkers can serve as surrogate markers of gut inflammation. [21]. For example, fecal calprotectin has become a clinical standard to assess IBD activity, predict relapse and monitor response to treatment [16]. A study conducted among adolescents has revealed that, on one hand, they tend not to report all of their symptoms and, on the other hand, they are not embarrassed by the idea of collecting their stool [17]. Thus, the stool test is appropriate and well received by this patient group. Although the study presented here was conducted with an adult population, the findings are consistent with those of the study conducted with adolescents. It indeed appears that the stool test was the test with which patients were the most comfortable and, even though it is the least-often explained by physicians, patients had a very good understanding of the need to undergo this test.

Colonoscopy, colon biopsy and medical imaging are procedures that are longer to perform and more invasive than a blood or stool test). Generally speaking, procedures raise more concerns about the results, the results increase concerns about the illness and physicians explain the reasons why they are needed to patients more frequently. A colonoscopy is often requested and very seldom refused. This is the procedure with which patients are the least comfortable. It is also the one that generates the most concerns about the results and whose results raise the most concerns about the illness. This perhaps explains why, of all the monitoring tools available, this is the procedure for which physicians most often provide explanations of why it is needed, the impact it will have on treatments, the inherent invasive nature of the procedure and the inherent potential risks. A colonoscopy is the gold standard procedure to assess disease activity but it is invasive and expensive and long to perform [22]. For patients, this procedure is demanding, as it requires motivation, planning and preparation (dietary restrictions and the need to take purgatives) [41]. In one study, 70% of respondents indicated they had not received enough information about the procedure, which decreased their comfort during the procedure [29]. A study on patient perceptions of the colonoscopy has shown a lack of knowledge about anatomy, the procedure and the reason for undergoing a colonoscopy [33]. These findings could explain why physicians more often explain to patients why this procedure is needed, as well as the impact the results will have on treatment and the potential risks associated with this procedure.

A biopsy during the colonoscopy is often orderded and very seldom refused. This is the procedure for which the reasons why it is needed are the least frequently explained to patients by their physicians and of which the presentation of the results generates the least concern about the illness. Patients nevertheless reported a high level of comfort with this procedure. However, patients with longer duration of disease usually are very concerned about the results of the biopsy because they are at higher risk of cancer. The goal of surveillance with colonosocopy is to reduce mortality and morbidity associated with colorectal cancer (CRC) by detecting asymptomatic cancers and premalignant lesions [24]. Ananthakrishnan, Cagan [26] have shown that the rate of CRC among IBD patients who had recently undergone a colonoscopy (within the previous 36 months) was lower and the mortality rate was lower for patients diagnosed with a CRC, which underscores the importance of adherence to surveillance colonoscopy, as was observed in this present study. Despite the risk of developing CRC, a colon biopsy remains the procedure for which patients receive explanations of why it is needed the least often and for which their understanding of why the procedure is needed is the lowest.

Among all of the procedures presented in this study, medical imaging is the least-often requested. It is more commonly refused than colonoscopy, colon biopsy, and stool test. This is the procedure for which patient level of comfort is the highest and for which the potential risks and the level of invasiveness are the least-often explained to patients. The additional information provided by medical imaging could change therapeutic decisions and have an impact on the clinical course of the illness, particularly in CD where it is most often ordered. [27]. Medical imaging techniques such as computerized tomography (CT) and magnetic resonance imaging (MRI) are increasingly used in the assessment of IBD [14]. A CT scan is an excellent imaging method for IBD, but the radiation doses are considerably higher than with other imaging methods. Given the chronic nature of IBD, patients are at risk of being exposed to an accumulation of potentially harmful ionizing radiation throughout their lifetime of medical follow-up, thereby increasing the risk of cancer among a population already at risk. Magnetic resonance imaging and small intestine contrast enhanced ultrasonography therefore emerge as radiation-free alternatives that provide results that compare to those of a CT scan in terms of accuracy [19]. No imaging technique is perfect, but each method plays a potential role in the assessment of IBD. Each method has its share of risks and benefits, and various aspects such as costs, exposure to radiation, the need for anesthesia and image quality must also be considered [27]. One study has shown that patients with aggressive lymphoma experienced high levels of anxiety during the period in which they had to undergo routine medical imaging scans to monitor their illness [32]. A systematic review of literature has been conducted to better understand the experience of patients as they undergo medical imaging procedures [34]. It has been shown that patients frequently have a negative experience that could stem directly from certain aspects of the procedure related to the production of high-quality images, such as: MRI noise; exposure to magnetism or radiation; holding one’s breath; the use of a contrast medium; and intestinal distention. As these elements help produce high-quality images, it is important that the reasons for these negative aspects be explained to patients. Yet, in the findings presented here, this is the procedure about which the fewest number of patients had received information about potential risks and the degree of invasiveness, and about which the patient level of comfort was highest.

This study may present certain limitations that could potentially limit the interpretation of the results. First, by virtue of confidentiality restrictions, participant selection was conducted through a questionnaire on the CCC website, which may represent a more motivated and engaged group of respondents. A higher response rate would have provided a larger sample size for broader application of study results.Second, the questions used to determine the reasons patients refused to undergo a test/procedure did not provide any clarifications as to the causes of the refusal, especially for the blood tests. An additional study should be conducted to shed light on the reasons that lead patients to refuse the test/procedure. With the latest advances in genomics, medical imaging and regenerative medicine, more precise diagnoses and personalized treatments have now become current fields of reseearch [49]. Thus, the development of genetic tests that could result in better diagnosis and patient response monitoring may lead to an improvement of their living conditions.

## Conclusions

In a patient-centered care approach, it is essential to gain a better understanding of patient perceptions of diagnostic and monitoring tests and procedures. Acceptance of the test or procedure by patients is essential for them to adhere to the monitoring process. Furthermore, if the anxiety generated by the test/procedure is too great or if the risks outweigh the benefits, the patient is at risk of refusing the test or procedure. The present study thus takes a first step in this direction and therefore provides findings that are useful to physicians. First, it has become clear that there is problem with the blood test. This test appears to be trivialized by physicians as few explanations are given to patients, who actually refuse this test in great numbers. The level of patient understanding of this test is in fact not very high. By being aware of this problem, physicians are in a better position to prevent patient refusal by modifying their practice, by providing more information about this test and by discussing it with their patients in order to understand their reluctance to undergo this test. In addition, since patients are very comfortable with the stool test, which offers a non-invasive and very revealing monitoring solution, physicians can turn to this test with patients who refuse the blood test. As procedures generate much concern among patients, physicians must take the time to properly explain not only the reasons for these procedures, but also the potential risks and the level of invasiveness of the ordered procedure. This study also clearly shows that the risks of having a false positive or negative are not sufficiently communicated to patients. It is therefore important that physicians transfer this information to their patients as part of their practice. Lastly, the theoretical contribution of this study is based on the dissociation between a strictly rational understanding of information and the tranfer of this information to the patient’s medical decision-making process that generates cognitive limitations and emotional factors, which in turn distorts the manner in which the information is processed by the patient. Thus, despite the transfer of information from physician to patient, the latter loses part of this information when the time has come to shift into action and/or make a decision, as the emotions, values and fears alter the course of the patient’s processing of the information.

## Additional file

Additional file 1: Online Questionnaire: Inflammatory Bowel Disease Patient Perceptions of Diagnostic and Monitoring Tests and Procedures. This online questionnaire was designed to better understand the concerns raised by the tests/procedures among IBD patients, the transfer of information from the physician to patients and the patients’ understanding of these tests/procedures, as well as the rate of prescriptions, the reasons patients refuse to undergo such tests/procedures and the sociodemographic profile of the participants (Additional file 1- Online Questionnaire). (DOC 179 kb)

## Abbreviations

CCC: Crohn’s and Colitis Canada
CD: Crohn’s disease
CRC: Colorectal cancer
CT: Computerized tomography
IBD: Inflammatory bowel disease
UC: Ulcerative colitis
