# Supplementary material for: Inflammatory bowel disease patient perceptions of diagnostic and monitoring tests and procedures
Source: BMC Gastroenterol. 2019 Feb 13;19:30. doi: 10.1186/s12876-019-0946-8 (PMC6374885; doi:10.1186/s12876-019-0946-8)
Supplement: Supplementary file 1 — Online Questionnaire: Inflammatory Bowel Disease Patient Perceptions of Diagnostic and Monitoring Tests and Procedures. This online questionnaire was designed to better understand the concerns raised by the tests/procedures among IBD patients, the transfer of information from the physician to patients and the patients’ understanding of these tests/procedures, as well as the rate of prescriptions, the reasons patients refuse to undergo such tests/procedures and the sociodemographic profile of the participants (Additional file 1- Online Questionnaire). (DOC 179 kb) [file 12876_2019_946_MOESM1_ESM.doc]

**Additional File 1**

**Online Questionnaire**

**Tests and procedures**

**This section concerns your latest test reports and/or other procedures regarding your Inflammatory Bowel Disease.**

Please answer yes or no to the following statement if you were prescribed any of these tests or procedures. Depending on your answers, further questions will appear on screen.

| **Analysis Test** | | |
| --- | --- | --- |
| General Blood test | No | Yes |
| Colonoscopy | No | Yes |
| Colon Biopsy | No | Yes |
| Medical Imagery (Abdominal Echography, Enteroscan, Entero-MRI) | No | Yes |
| Stool test | No | Yes |

If you answer **yes**:

Please indicate: if you understand the reason why this test/procedure was requested = 1 (you did not understand why) to 6 (you completely understand why)

Please indicate: if you were 1 (not comfortable to undergo the test) to 6 (completely comfortable to undergo this test)

If you answered **No**: please indicate the reason for your refusal (to undergo the test) according to the following scale = 1 (not at all for this reason) to 6 (refused 100% for this reason)

| **General blood Test** | | | | | | |
| --- | --- | --- | --- | --- | --- | --- |
| **If yes:** 1 = (not at all comfortable to undergo this test) to 6 (completely comfortable to undergo this test) | 1 | 2 | 3 | 4 | 5 | 6 |
| **If no because** : 1 = (not at all for this reason) to 6 (refused 100% for this reason) |  |  |  |  |  |  |
| Time |  |  |  |  |  |  |
| Pain |  |  |  |  |  |  |
| Cost |  |  |  |  |  |  |
| Potential Risk |  |  |  |  |  |  |
| Side effect |  |  |  |  |  |  |
| Fear of the results |  |  |  |  |  |  |
| Test too revealing (fear of finding other problems) |  |  |  |  |  |  |
| Confidentiality |  |  |  |  |  |  |
| When your physician proposed this test did he explain why it was needed? | No | | | Yes | | |
| What is your level of understanding of the reasons why this test was requested? | 1 | 2 | 3 | 4 | 5 | 6 |
| When your IDB specialist prescribed this test, did he inform you: |  | | |  | | |
| Of the impact that each test result may have on the treatment? | No | | | Yes | | |
| That there may be a false positive, false negative? | No | | | Yes | | |
| Of the level of invasiveness? | No | | | Yes | | |
| Of the potential risks? | No | | | Yes | | |
| Proposed alternative tests? | No | | | Yes | | |
| Did the results of this test increase your preoccupation with your disease? | No | | | Yes | | |
| Please evaluate your level of preoccupation at the moment you were presented with the results of this test. | 1 | 2 | 3 | 4 | 5 | 6 |
| What was your level of understanding of the potential treatments? | 1 | 2 | 3 | 4 | 5 | 6 |

| **Colonoscopy** | | | | | | |
| --- | --- | --- | --- | --- | --- | --- |
| **If yes:** 1 = (not at all comfortable to undergo this test) to 6 (completely comfortable to undergo this test) | 1 | 2 | 3 | 4 | 5 | 6 |
| **If no because** : 1 = (not at all for this reason) to 6 (refused 100% for this reason) |  |  |  |  |  |  |
| Time |  |  |  |  |  |  |
| Pain |  |  |  |  |  |  |
| Cost |  |  |  |  |  |  |
| Potential Risk |  |  |  |  |  |  |
| Side effect |  |  |  |  |  |  |
| Fear of the results |  |  |  |  |  |  |
| Test too revealing (fear of finding other problems) |  |  |  |  |  |  |
| Confidentiality |  |  |  |  |  |  |
| When your physician proposed this test did he (she) explain why it was needed? | No | | | Yes | | |
| What is your level of understanding of the reasons why this test was requested? | 1 | 2 | 3 | 4 | 5 | 6 |
| When your IDB specialist prescribed this test, did he (she) inform you: |  | | |  | | |
| Of the impact that each result may have on the treatment? | No | | | Yes | | |
| That there may be a false positive, false negative? | No | | | Yes | | |
| On the level of invasiveness? | No | | | Yes | | |
| Of the potential risks? | No | | | Yes | | |
| Proposed alternative tests? | No | | | Yes | | |
| Did the results of this test increase your preoccupation with your disease? | No | | | Yes | | |
| Please evaluate your level of preoccupation at the moment you were presented with the results of this test. | 1 | 2 | 3 | 4 | 5 | 6 |
| What was your level of understanding of the potential treatments? | 1 | 2 | 3 | 4 | 5 | 6 |

| **Colon Biopsy** | | | | | | |
| --- | --- | --- | --- | --- | --- | --- |
| **If yes:** 1 = (not at all comfortable to undergo this test) to 6 (completely comfortable to undergo this test) | 1 | 2 | 3 | 4 | 5 | 6 |
| **If no because** : 1 = (not at all for this reason) to 6 (refused 100% for this reason) |  |  |  |  |  |  |
| Time |  |  |  |  |  |  |
| Pain |  |  |  |  |  |  |
| Cost |  |  |  |  |  |  |
| Potential Risk |  |  |  |  |  |  |
| Side effect |  |  |  |  |  |  |
| Fear of the results |  |  |  |  |  |  |
| Test too revealing (fear of finding other problems) |  |  |  |  |  |  |
| Confidentiality |  |  |  |  |  |  |
| When your physician proposed this test did he (she) explain why it was needed? | No | | | Yes | | |
| What is your level of understanding of the reasons why this test was requested? | 1 | 2 | 3 | 4 | 5 | 6 |
| When your IDB specialist prescribed this test, did he (she) inform you: |  | | |  | | |
| Of the impact that each result may have on the treatment? | No | | | Yes | | |
| That there may be a false positive, false negative? | No | | | Yes | | |
| Of the level of invasiveness? | No | | | Yes | | |
| Of the potential risks? | No | | | Yes | | |
| Proposed alternative tests? | No | | | Yes | | |
| Did the results of this test increase your preoccupation with your disease? | No | | | Yes | | |
| Please evaluate your level of preoccupation at the moment you were presented with the results of this test. | 1 | 2 | 3 | 4 | 5 | 6 |
| What was your level of understanding of the potential treatments? | 1 | 2 | 3 | 4 | 5 | 6 |

| **Medical Imagery (Abdominal Echography, Enteroscan, Entero-MRI)** | | | | | | |
| --- | --- | --- | --- | --- | --- | --- |
| **If yes:** 1 = (not at all comfortable to undergo this test) to 6 (completely comfortable to undergo this test) | 1 | 2 | 3 | 4 | 5 | 6 |
| **If no because** : 1 = (not at all for this reason) to 6 (refused 100% for this reason) |  |  |  |  |  |  |
| Time |  |  |  |  |  |  |
| Pain |  |  |  |  |  |  |
| Cost |  |  |  |  |  |  |
| Potential Risk |  |  |  |  |  |  |
| Side effect |  |  |  |  |  |  |
| Fear of the results |  |  |  |  |  |  |
| Test too revealing (fear of finding other problems) |  |  |  |  |  |  |
| Confidentiality |  |  |  |  |  |  |
| When your physician proposed this test did he (she) explain why it was needed? | No | | | Yes | | |
| What is your level of understanding of the reasons why this test was requested? | 1 | 2 | 3 | 4 | 5 | 6 |
| When your IDB specialist prescribed this test, did he (she) inform you: |  | | |  | | |
| Of the impact that each result may have on the treatment? | No | | | Yes | | |
| That there may be a false positive, false negative? | No | | | Yes | | |
| Of the level of invasiveness? | No | | | Yes | | |
| Of the potential risks? | No | | | Yes | | |
| Proposed alternative tests? | No | | | Yes | | |
| Did the results of this test increase your preoccupation with your disease? | No | | | Yes | | |
| Please evaluate your level of preoccupation at the moment you were presented with the results of this test. | 1 | 2 | 3 | 4 | 5 | 6 |
| What was your level of understanding of the potential treatments? | 1 | 2 | 3 | 4 | 5 | 6 |

| **Stool test** | | | | | | |
| --- | --- | --- | --- | --- | --- | --- |
| **If yes:** 1 = (not at all comfortable to undergo this test) to 6 (completely comfortable to undergo this test) | 1 | 2 | 3 | 4 | 5 | 6 |
| **If no because** : 1 = (not at all for this reason) to 6 (refused 100% for this reason) |  |  |  |  |  |  |
| Time |  |  |  |  |  |  |
| Pain |  |  |  |  |  |  |
| Cost |  |  |  |  |  |  |
| Potential Risk |  |  |  |  |  |  |
| Side effect |  |  |  |  |  |  |
| Fear of the results |  |  |  |  |  |  |
| Test too revealing (fear of finding other problems) |  |  |  |  |  |  |
| Confidentiality |  |  |  |  |  |  |
| When your physician proposed this test did he (she) explain why it was needed? | No | | | Yes | | |
| What is your level of understanding of the reasons why this test was requested? | 1 | 2 | 3 | 4 | 5 | 6 |
| When your IDB specialist prescribed this test, did he (she) inform you: |  | | |  | | |
| Of the impact that each result may have on the treatment? | No | | | Yes | | |
| That there may be a false positive, false negative? | No | | | Yes | | |
| Of the level of invasiveness? | No | | | Yes | | |
| Of the potential risks? | No | | | Yes | | |
| Proposed alternative tests? | No | | | Yes | | |
| Did the results of this test increase your preoccupation with your disease? | No | | | Yes | | |
| Please evaluate your level of preoccupation at the moment you were presented with the results of this test. | 1 | 2 | 3 | 4 | 5 | 6 |
| What was your level of understanding of the potential treatments? | 1 | 2 | 3 | 4 | 5 | 6 |

**Information for statistical purposes**

In which province or territory do you live?

Alberta

British-Colombia

Prince-Edward Island

Manitoba

New-Brunswick

Nova-Scotia

Nunavut

Ontario

Québec

Saskatchewan

Newfoundland and Labrador

North-West territories

Yukon

What are the first three numbers/letters from you postal code? ___ ____ ___

Are you a :

Woman ___ Man ___

What is your age group:

18 to 24 years___

25 to 34 years ___

35 to 44 years ___

45 to 54 years ___

55 to 64 years ___

65 to 74 years ___

75 to 84 years ___

85 years and more ___

What is your civil status?

Married/common law_____

Divorced/separated____

Widow/widower ____

Single_____

What is the highest level of education that you have completed?

Elementary ___

High School ___

Professional or College ___

Undergraduate University degree ___

Graduate University degree ___

Are-you a :

Smoker ____ Non Smoker____

Please indicate your current general health status:

Very Bad ___

Bad ___

O.K. ___

Well ___

Very Well___

What type of Inflammatory Bowel Disease do you have ?

Ulcerative Colitis (UC) ____ Crohn’s disease (CD) ____

How long have you had an Inflammatory Bowel Disease (years ou months)? ___

How many times a year do you consult your IDB specialist? ____

In the last two years, how would you describe the symptoms caused by your Inflammatory Bowel Disease?

No symptoms ___

Low Symptoms (no effect on daily activities) ___

Medium Symptoms (either they impact on your daily activities and they may require that you take sick leave) ___

Acute Symptoms (either they prevent you from participating in you daily activities, or you are on sick leave or you are hospitalised) ___

What treatment regimen do you currently follow?

ASA ____

Antibiotic___

Immunosupressors (including Azathioprine, Mercaptopurine, Mexthotrexate) ________

Biological Agents (including Infliximab, Adalimumab)____________

No treatment ____
